# The surfactant protein C mutation A116D alters cellular processing, stress tolerance, surfactant lipid composition, and immune cell activation

**DOI:** 10.1186/1471-2466-12-15

**Published:** 2012-03-29

**Authors:** Ralf Zarbock, Markus Woischnik, Christiane Sparr, Tobias Thurm, Sunčana Kern, Eva Kaltenborn, Andreas Hector, Dominik Hartl, Gerhard Liebisch, Gerd Schmitz, Matthias Griese

**Affiliations:** 1 Childrens' Hospital of the Ludwig-Maximilians-University, Lindwurmstr. 4, 80337 Munich, Germany; 2 University of Regensburg, Institute for Clinical Chemistry and Laboratory Medicine, Franz-Josef-Strauss-Allee 11, 93053 Regensburg, Germany

## Abstract

**Background:**

Surfactant protein C (SP-C) is important for the function of pulmonary surfactant. Heterozygous mutations in *SFTPC*, the gene encoding SP-C, cause sporadic and familial interstitial lung disease (ILD) in children and adults. Mutations mapping to the BRICHOS domain located within the SP-C proprotein result in perinuclear aggregation of the proprotein. In this study, we investigated the effects of the mutation A116D in the BRICHOS domain of SP-C on cellular homeostasis. We also evaluated the ability of drugs currently used in ILD therapy to counteract these effects.

**Methods:**

SP-C^A116D ^was expressed in MLE-12 alveolar epithelial cells. We assessed in vitro the consequences for cellular homeostasis, immune response and effects of azathioprine, hydroxychloroquine, methylprednisolone and cyclophosphamide.

**Results:**

Stable expression of SP-C^A116D ^in MLE-12 alveolar epithelial cells resulted in increased intracellular accumulation of proSP-C processing intermediates. SP-C^A116D ^expression further led to reduced cell viability and increased levels of the chaperones Hsp90, Hsp70, calreticulin and calnexin. Lipid analysis revealed decreased intracellular levels of phosphatidylcholine (PC) and increased lyso-PC levels. Treatment with methylprednisolone or hydroxychloroquine partially restored these lipid alterations. Furthermore, SP-C^A116D ^cells secreted soluble factors into the medium that modulated surface expression of CCR2 or CXCR1 receptors on CD4^+ ^lymphocytes and neutrophils, suggesting a direct paracrine effect of SP-C^A116D ^on neighboring cells in the alveolar space.

**Conclusions:**

We show that the A116D mutation leads to impaired processing of proSP-C in alveolar epithelial cells, alters cell viability and lipid composition, and also activates cells of the immune system. In addition, we show that some of the effects of the mutation on cellular homeostasis can be antagonized by application of pharmaceuticals commonly applied in ILD therapy. Our findings shed new light on the pathomechanisms underlying SP-C deficiency associated ILD and provide insight into the mechanisms by which drugs currently used in ILD therapy act.

## Background

Pulmonary surfactant is a phospholipid/protein mixture secreted to the alveolar surface by alveolar type 2 (AT2) cells [1]. It reduces surface tension and prevents alveolar collapse at the end of expiration [2]. A normal composition and homeostasis of pulmonary surfactant is critical for its surface-tension-reducing properties and gas exchange in the alveoles of the lung. Surfactant protein C (SP-C) is a hydrophobic, lung-specific protein that coisolates with the phospholipid fraction of pulmonary surfactant [3]. SP-C is synthesized exclusively by AT2 cells as a 197 amino acid proprotein (proSP-C) and proteolytically processed into the 4.2 kDa mature protein by a sequence of proteolytic cleavages [4]. Mature SP-C is subsequently secreted together with lipids and other surfactant components to the alveolar surface [3,5]. AT2 cells contain specialized lysosome-derived organelles for the storage of surfactant prior to its secretion. Exocytosis is facilitated by fusion of these so-called lamellar bodies (LBs) with the plasma membrane [6]. The SNARE proteins syntaxin 2 and SNAP-23 are associated with the plasma membrane and to some degree with lamellar bodies and have been shown to be required for regulated surfactant secretion [7,8].

Interstitial lung diseases (ILD) are a heterogeneous group of respiratory disorders that can be classified into those with known and unknown etiologies [9]. ILD are characterized by deposition of cellular and non-cellular components into the lung parenchyma. They vary widely in regard to radiological presentation, histopathological features, and clinical course [10]. ILD are mostly chronic and associated with high morbidity and mortality. Typical features of ILD include dyspnoea, the presence of diffuse infiltrates on chest radiographs and abnormal pulmonary function tests with evidence of a restrictive ventilatory defect and/or impaired gas exchange [11].

Mutations in the surfactant protein genes *SFTPB *and *SFTPC *as well as in the ABC-transporter coding gene *ABCA3*, all of them resulting in a disturbed lung surfactant homeostasis, have been identified as genetic causes in some forms of ILD [12-16]. While loss-of-function mutations in SP-B result in surfactant deficiency and fatal neonatal lung disease, consequences of mutations in SP-C tend to be less severe, ranging from fatal pulmonary surfactant deficiency to childhood ILD [17]. Most SP-C mutations cluster within the preprotein's BRICHOS domain and lead to misfolding of the preprotein, aberrant trafficking and processing [3]. To date, all affected individuals with BRICHOS domain mutations have been heterozygous with no detectable mature SP-C in their lungs, suggesting a dominant-negative effect of the mutant allele [3,12]. Moreover, in cell lines expressing BRICHOS domain mutations, proSP-C forms perinuclear aggregates, consistent with the cell's inability to clear aggregates of misfolded protein and a toxic gain-of-function [12,18]. Accumulation of misfolded proSP-C may trigger several distinct pathological mechanisms, such as induction of endoplasmic reticulum (ER) stress, cytotoxicity, and caspase 3- and caspase 4-mediated apoptosis [14,19,20]. These factors might contribute to ILD by causing cell injury and apoptotic death of AT2 cells.

Current treatment of ILD in children is unfortunately empirical. Since an inflammatory component is present in ILD, corticosteroids and immunosuppressive drugs like azathioprine are used in ILD therapy [21]. Corticosteroids are anti-inflammatory and stimulate surfactant protein transcription [22,23]. While chloroquine and its less toxic derivative hydroxychloroquine are also used in ILD treatment, their mode of action remains controversial [21,24]. It has been proposed that chloroquine acts on lysosomal function or stimulates the generation of lamellar bodies [25,26]. Thus, there is obviously an urgent need to define the target mechanism of the treatments currently applied in ILD therapy.

Cell chaperones which assist in normal protein folding and removal of misfolded proteins may pose promising therapeutic targets in ILD [27]. For example, the *SFTPC *mutation Δexon4 leads to accumulation of misfolded SP-C and a subsequent upregulation the major ER chaperone GRP78/BiP in an attempt to maintain surfactant biosynthesis in the presence of ER stress [19]. Pharmacological intervention in order to increase the expression of GRP78 or other chaperones, like Hsp90, Hsp70, calreticulin and calnexin, may be suitable to counteract the deleterious effects of the accumulation of aberrant protein in the cells.

We hypothesized that pharmaceuticals currently used in ILD therapy may operate by counteracting disturbances of cellular homeostasis caused for example by mutations in SP-C. Therefore, the aim of the present study was to investigate the intracellular alterations in alveolar epithelial cells expressing SP-C^A116D ^and the ability of pharmaceuticals commonly used in ILD therapy to modulate the effects caused by mutant SP-C. The A116D mutation was chosen as a representative of the BRICHOS domain mutations. It was described at first in a boy with severe respiratory distress and a diagnosis of nonspecific interstitial pneumonitis [28]. We studied the impact of the A116D mutation on proSP-C processing, cellular stress tolerance, lipid composition, and immunity. In addition, we investigated modulation of the cellular pathomechanisms by pharmaceutical drugs currently applied in ILD therapy.

## Methods

### Plasmid vectors

Eukaryotic expression vectors containing the full human *SFTPC *gene fused to either EGFP-tag (pEGFP-N1/hSP-C1-197 and pEGFP-C1/hSP-C1-197 to obtain proSP-C with EGFP fused to the C- or N-terminus, respectively) or hemagglutinin (HA)-tag (proSP-C with N-terminal HA-tag) were obtained as previously described [12]. The A116D point mutation was introduced into the wild-type (WT) *SFTPC *gene in these vectors using the QuickChange site-directed mutagenesis kit (Stratagene, La Jolla, USA) and the following primers: A116D_forward: 5'-GCC TAC AAG CCA GAC CCT GGC ACC TGC-3', A116D_reverse: 5'-GCA GGT GCC AGG GTC TGG CTT GTA GGC-3', following the manufacturer's protocol. The successful mutagenesis was confirmed by Sanger sequencing.

### MLE-12 cell lines and transfection

The mouse MLE-12 lung epithelial cell line (CRL-2119) [29] was obtained from the American Type Culture Collection (ATCC) and maintained in RPMI medium supplemented with 10% fetal bovine serum. Cells were transfected using FuGene 6 (Roche, Penzberg, Germany) according to the manufacturer's instructions. MLE-12 cells stably transfected with either pcDNA3/HA-hSP-C1-197 or pcDNA3/HA-hSP-CA116D vectors were obtained by selecting transfected cells in the presence of 600 μg/ml G418 in RPMI medium for four weeks. For drug exposure experiments, stable cells were grown for 24 hours in the presence of 10 μM of cyclophosphamide, azathioprine, methylprednisolone or hydroxychloroquine. Untreated cells that were cultured in parallel served as controls.

### Immunoblotting

Total cell proteins were obtained by lysing the cells in lysis buffer [PBS, 20 mM EDTA, 1% v/v Elugent (Calbiochem, Bad Soden, Germany), protease inhibitor (Complete; Roche, Manheim, Germany)]. For immunoblotting, 30 μg protein was separated under reducing conditions using 10% NuPage Bis-Tris gels (Invitrogen, Karlsruhe, Germany) and transferred to a PVDF membrane. The following primary antibodies were used: monoclonal rat anti-HA-tag (1:1000; Roche), monoclonal mouse anti-GFP (1:500; Clontech, Heidelberg, Germany), and polyclonal goat anti-calnexin (1:500), polyclonal goat anti-calreticulin (1:500), monoclonal mouse anti-HSP90α/β, polyclonal goat anti-HSP70 (1:1000), and monoclonal anti-β-actin HRP conjugate (1:10000) (all from Santa Cruz Biotechnology, Santa Cruz, CA). Signal was detected using chemiluminescent labeling with Amersham ECL Detection Reagents (GE Healthcare), densitometrically quantified and normalized to the β-actin signal.

### Immunofluorescence

24 hours after transfection, cells grown on coverslips were fixed with 4% paraformaldehyde, permeabilised with 10% Triton X-100, and blocked for 30 min in PBS with 5% FBS. The following primary antibodies were used in 1:200 dilution: polyclonal rabbit anti-mouse LAMP3 (Santa Cruz), monoclonal mouse anti-human CD63/LAMP3 (Chemicon, Schwalbach, Germany), polyclonal rabbit anti-EEA1 (Acris Antibodies, Herford, Germany), monoclonal mouse anti-ubiquitin (Biomol, Hamburg, Germany) and polyclonal rabbit anti-syntaxin 2 (Synaptic Systems, Berlin, Germany). Species specific Alexa Fluor 488 or Alexa Fluor 555 secondary antibodies (Invitrogen) were used at 1:200. Samples were mounted and Alexa Fluor or GFP fluorescence was examined with Axiovert 135 fluorescent microscope and evaluated with AxioVision 4.7.1 software (Carl Zeiss, Jena, Germany).

### Lactate dehydrogenase (LDH) assay

LDH activity in cell lysates and culture supernatants was determined using the method of Decker and Lohmann-Matthes [30]. Briefly, 100 μl sample was mixed with 30 μl dye solution (18 mg/ml *L*-lactate, 1 mg/ml iodonitrotetrazolium in PBS). After adding 15 μl of the catalyst (3 mg/ml NAD^+^, 2.3 mg/ml diaphorase, 0.03% BSA, 1.2% sucrose in PBS), absorbance at 492 nm was determined at one minute intervals for 15 minutes at 37°C. Absolute LDH activity was calculated from a standard curve, using purified LDH (Sigma, Munich, Germany). The lower limit of detection was 20 Units/l; the assay was linear to 2500 Units/l.

### Mass spectrometric lipid analysis

For lipid analysis, cells grown in Petri dishes were harvested by scraping off in 2 ml PBS supplemented with protease inhibitor (Complete, Roche). The cell suspension was then sonicated (four strokes, 10 seconds; Branson Digital Sonifier S450D). Lipid classes and subspecies were determined by electrospray ionization tandem mass spectrometry (ESI-MS/MS) using direct flow injection analysis, as described previously [31,32]. Cells were extracted according to the method described by Bligh and Dyer [33] in the presence of non-naturally occurring lipid species used as internal standards. A precursor ion scan of m/z 184 specific for phosphocholine containing lipids was used for phosphatidylcholine (PC), sphingomyelin (SPM) [32] and lysophosphatidylcholine (LPC) [31]. Neutral loss scans of m/z 141 and m/z 185 were used for phosphatidylethanolamine (PE) and phosphatidylserine (PS), respectively [34]. Phosphatidylglycerol (PG) was analyzed using a neutral loss scan of m/z 189 of ammonium adduct ions [35]. Ceramide and glucosylceramide were analyzed as previously described [36] using N-heptadecanoyl-sphingosine as internal standard. Quantification was achieved by calibration lines generated by addition of naturally occurring lipid species to pooled cell homogenate. All lipid classes were quantified with internal standards belonging to the same lipid class, except SM (PC internal standards). Each lipid class was calibrated with a variety of species covering chain lengths and number of double bonds of naturally occurring species. Correction of isotopic overlap of lipid species and data analysis was performed by self-programmed Excel macros for all lipid classes according to the described principles [32].

### Flow cytometry

Human lymphocytes and neutrophils were isolated from whole blood using LeucoSep (Greiner Bio-One, Solingen-Wald, Germany) and Ficoll-Isopaque gradient density isolation method (GE Healthcare) according to the manufacturer's instructions. Cells were incubated for 6 hours (neutrophils) or 24 hours (lymphocytes) at 37°C with supernatants of MLE-12 cells expressing wild-type or mutant proSP-C. Cell-free supernatants were collected after 48 hours of growth and concentrated 7-fold, using Microsep 1 k centrifugal concentrators (Millipore, Schwalbach, Germany). Cells were analyzed by four-colour flow cytometry (FACSCalibur; BD Biosciences, Heidelberg, Germany) as described previously [37]. The following antibodies were used: PE-conjugated mouse anti-human CCR2-B (R&D Systems, Minneapolis, USA), FITC labeled anti-human CD8, FITC labeled anti-human CD4, PE-conjugated mouse anti-human CD11b/Mac-1, and PE-conjugated mouse anti-human CD181 (CXCR1, IL-8RA) (all BD Biosciences Pharmingen). Results are presented as mean fluorescence intensity (MFI) after subtracting background binding provided by non-specific isotypes. Calculations were performed with CellQuest analysis software (BD Biosciences).

### Statistical methods

The results are given as means ± standard error (SE) of the individual number of different subjects, each individual value representing the mean of 3-4 determinations or as indicated. In the case of lipid analysis, the results are presented as means ± standard deviation. For the comparison of two groups, unpaired t-test or Mann-Whitney test were used where appropriate. Comparisons of multiple groups were made using ANOVA followed by Dunnett's post hoc multiple comparisons test. For correlations, Spearman's non-parametric test was used. P-values of less than 0.05 were considered as statistically significant. All tests were performed using GraphPad Prism 4.0 (GraphPad Software).

## Results

### MLE-12 cells process proSP-C^A116D ^differently from proSP-C^WT ^and accumulate proSP-C^A116D ^processing intermediates

**Figure 1:**
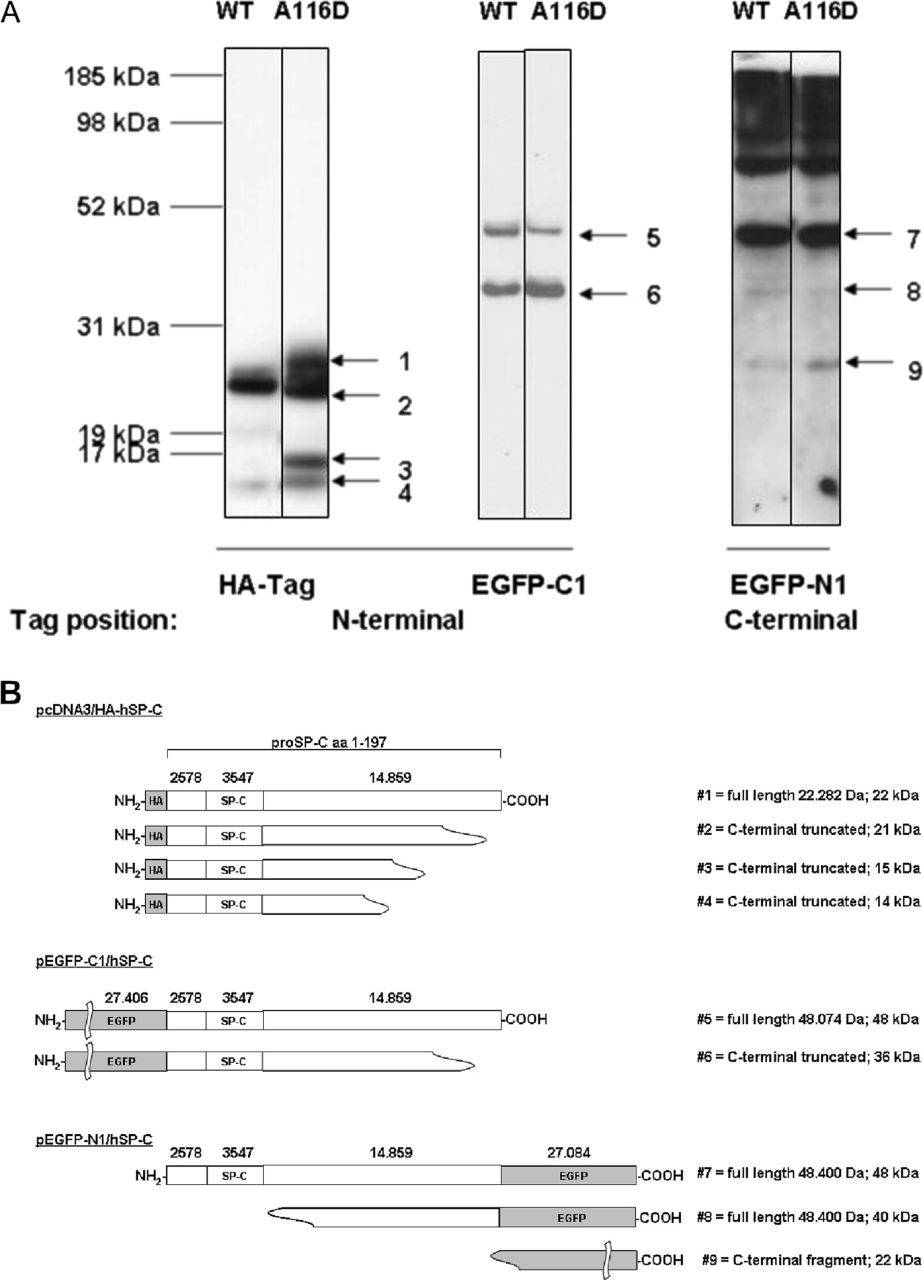
**Processing features of proSP-C^WT ^or proSP-C^A116D^**. (A) Immunoblotting of total cell lysates with tag-specific antibodies. Cell lysates obtained from MLE-12 cells stably expressing fusion protein of proSP-C with an N-terminal HA-tag (left panel), transiently transfected cells expressing fusion protein of proSP-C with an N-terminal EGFP (EGFP-C1, middle panel) or a C-terminal EGFP (EGFP-N1, right panel), present with several bands corresponding to different proSP-C processing intermediates, in which the tag sequence is retained. (B) Based on the size of the bands, the projected corresponding intermediate species of the fusion constructs are depicted. The cleavage sites are only estimates due to the limited resolution of the technique. EGFP-C1 (band #5) and EGFP-N1 (band #9) are expressed as a full-length product of 48 kDa, HA-SP-C (band #1) of size 22 kDa.

To identify the intracellular processing intermediates of proSP-C, MLE-12 cells were transfected with eukaryotic expression vectors, allowing expression of fusion proteins between proSP-C and either an EGFP- or a HA-Tag. Stable expression of HA-tagged proSP-C^WT ^resulted in the appearance of a strong band at approximately 21 kDa and weaker bands at 22 kDa, 19 kDa, and 14 kDa (Figure 1A left). ProSP-C^A116D ^yielded bands similar to the wild type at 21 kDa and 14 kDa. We also observed a much stronger band at 22 kDa and a band at 15 kDa that was not seen in the wild type, indicating accumulation of proSP-C^A116D ^forms (Figure 1A, left). The postulated processing products are depicted in Figure 1B. Mature SP-C was never detectable because of the loss of the protein tag due to the final processing steps at the N-terminus.

Transient expression of N-terminal (C1) and C-terminal (N1) EGFP fusion products was detectable 24 hours post transfection. The primary N- and C-terminal fusion proteins were visible as bands at 48 kDa as expected. In the case of N-terminally tagged protein, a second band was seen at 36 kDa (Figure 1A, middle). There were no differences regarding band pattern between proSP-C^WT ^and proSP-C^A116D^. Likewise, no differences in band pattern between proSP-C^WT ^and proSP-C^A116D ^were seen for processing intermediates containing the C-terminal EGFP-tag (Figure 1A right). This suggested that there was no change in the cleavage pattern or kinetics regarding the truncation of proSP-C from the C-terminus, which is supposed to be the first cleavage step [4]. The lowest band corresponded to the EGFP-tag, which has a size of approximately 27 kDa.

### ProSP-C^A116D ^localizes to different intracellular compartments than proSP-C^WT^

**Figure 2:**
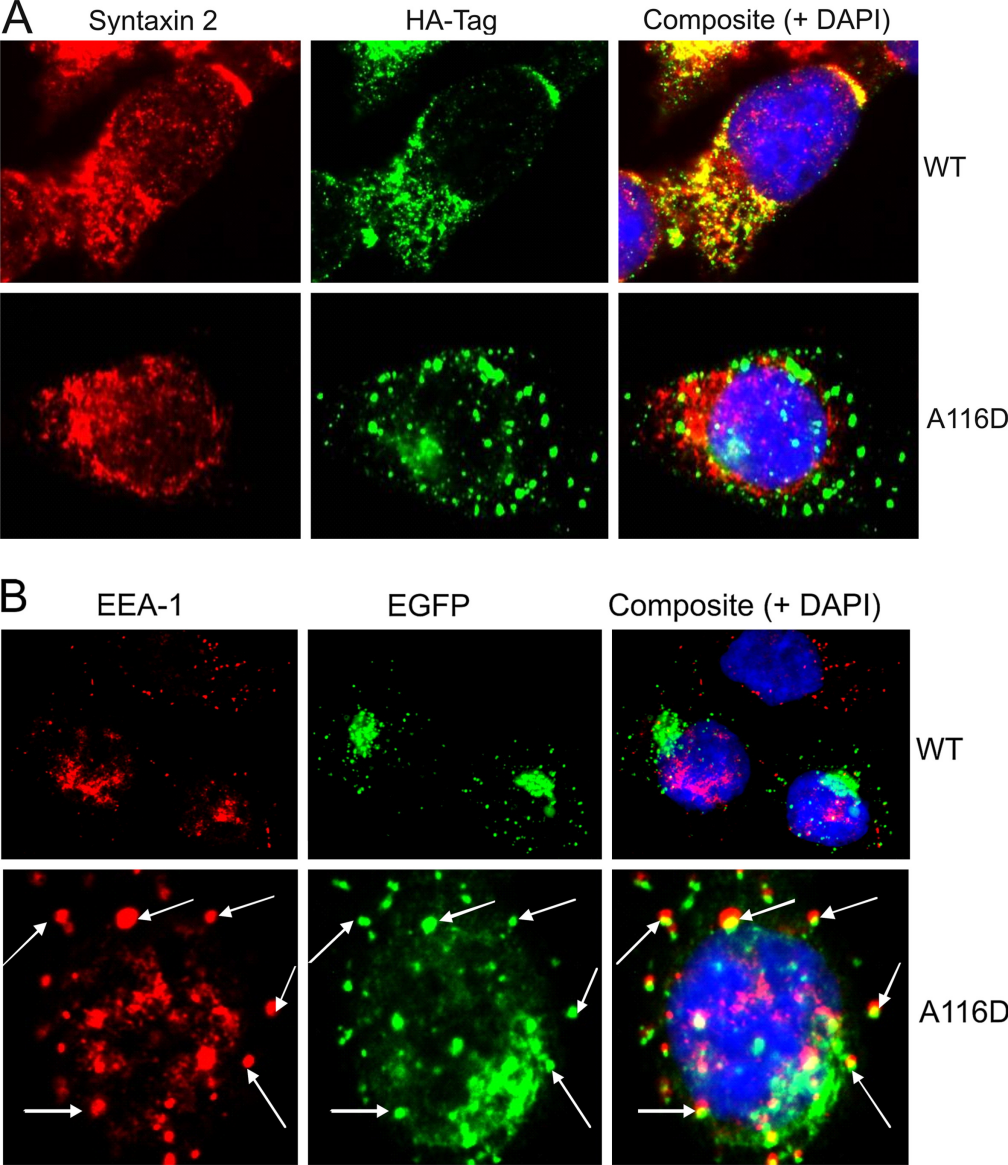
**Intracellular localization of proSP-C^WT ^and proSP-C^A116D ^forms in stabile MLE-12 cells**. Immunofluorescent analysis with an antibody against (A) EEA1 (red) and (B) syntaxin 2 (red) shows that while proSP-C^WT ^localized with syntaxin 2, a protein found within lamellar bodies as surfactant secretory vesicles, colocalization of proSP-C^A116D ^with syntaxin 2 was merely absent. ProSP-CA116D instead localized with in EEA1 which did not show any colocalization with proSP-C^WT^. Nuclei are stained with DAPI (blue).

The intracellular localization of preprotein species, monitored by immunofluorescence, differed between MLE-12 cells stably expressing proSP-C^WT ^or proSP-C^A116D^. While HA-tagged proSP-C^WT ^colocalized well with syntaxin 2, proSP-C^A116D ^did not (Figure 2A). In contrast, EGFP-tagged proSP-C^A116D ^was partially present in early endosomes detected as EEA1-positive vesicles, while proSP-C^WT ^was almost absent in those compartments (Figure 2B), thus confirming previous data [38]. Again, with this approach mature SP-C was not detectable because of the loss of the tag due to processing.

### Expression of SP-C^A116D ^increases susceptibility of MLE-12 cells to exogenous stress imposed by pharmacological substances

**Figure 3:**
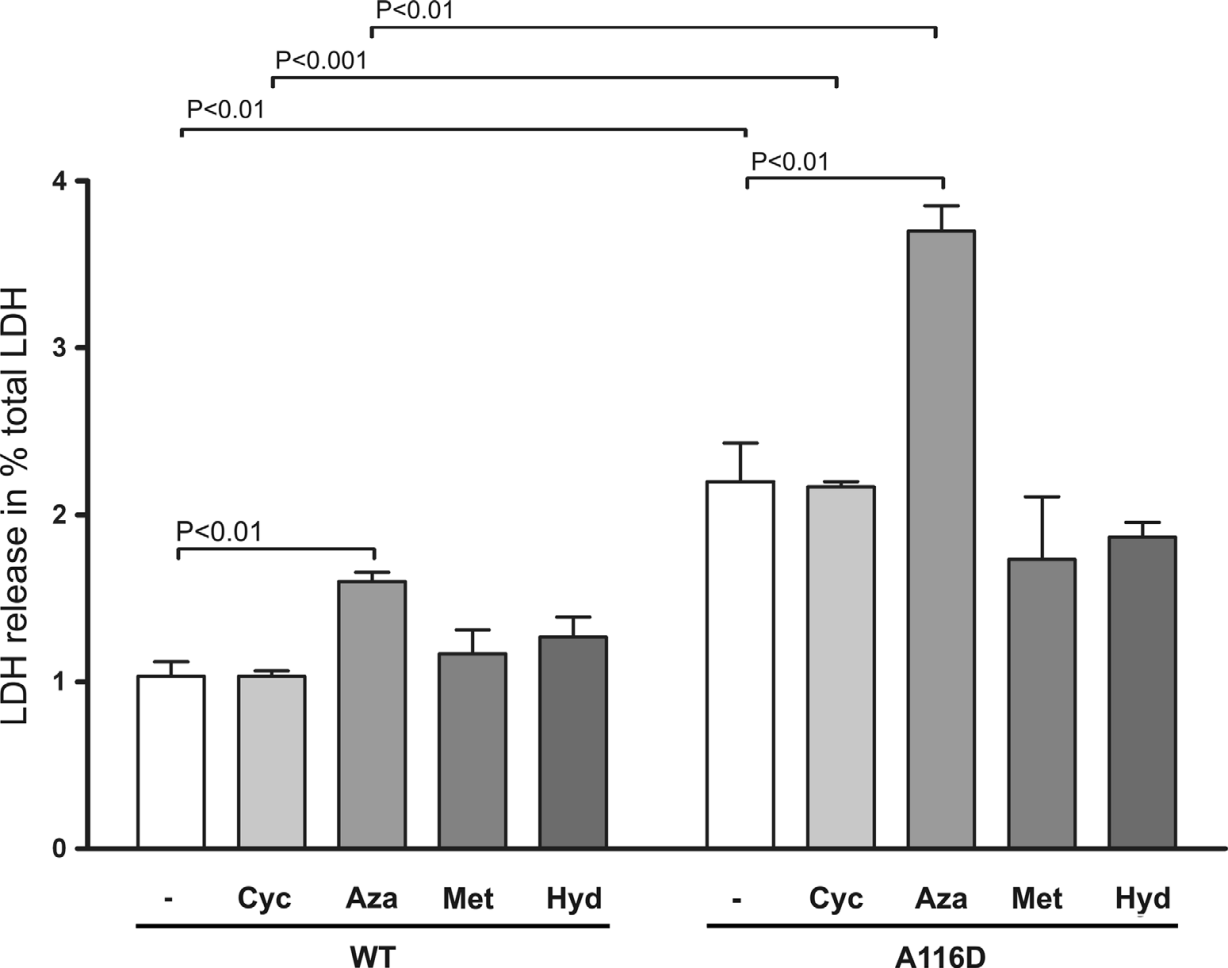
**Viability of MLE12 cells expressing SP-C^WT ^or SP-C^A116D ^before and after treatment with drugs used in therapy**. MLE-12 cells stably expressing SP-C^WT ^or SP-C^A116D ^were incubated for 24 hours with 10 μM each of cyclophosphamide (+Cyc), azathioprine (+Aza), methylprednisolone (+Met), or hydroxychloroquine (+Hyd). LDH release, a sign of decreased cell fitness, of treated vs. untreated (-) cells is expressed as % of total LDH. Only significant p-values are depicted.

In order to determine the stress level of cells expressing SP-C^A116D^, lactate dehydrogenase (LDH) release of stably transfected cells was measured. Expression of SP-C^A116D ^led to a marked overall increase of LDH release, compared to WT cells, suggesting a reduction of cell viability (Figure 3). Exposure of MLE-12 cells expressing SP-C^WT ^to pharmacological substances currently applied in ILD therapy significantly increased the release of LDH by the cells in the case of azathioprine. While azathioprine treatment resulted in a pronounced increase in LDH release also in cells expressing SP-C^A116D^, hydroxychloroquine, methylprednisolone and cyclophosphamide did not significantly alter LDH release by these cells.

### Modulation of chaperone level in cells expressing SP-C^WT ^and SP-C^A116D ^by pharmacological substances

**Figure 4:**
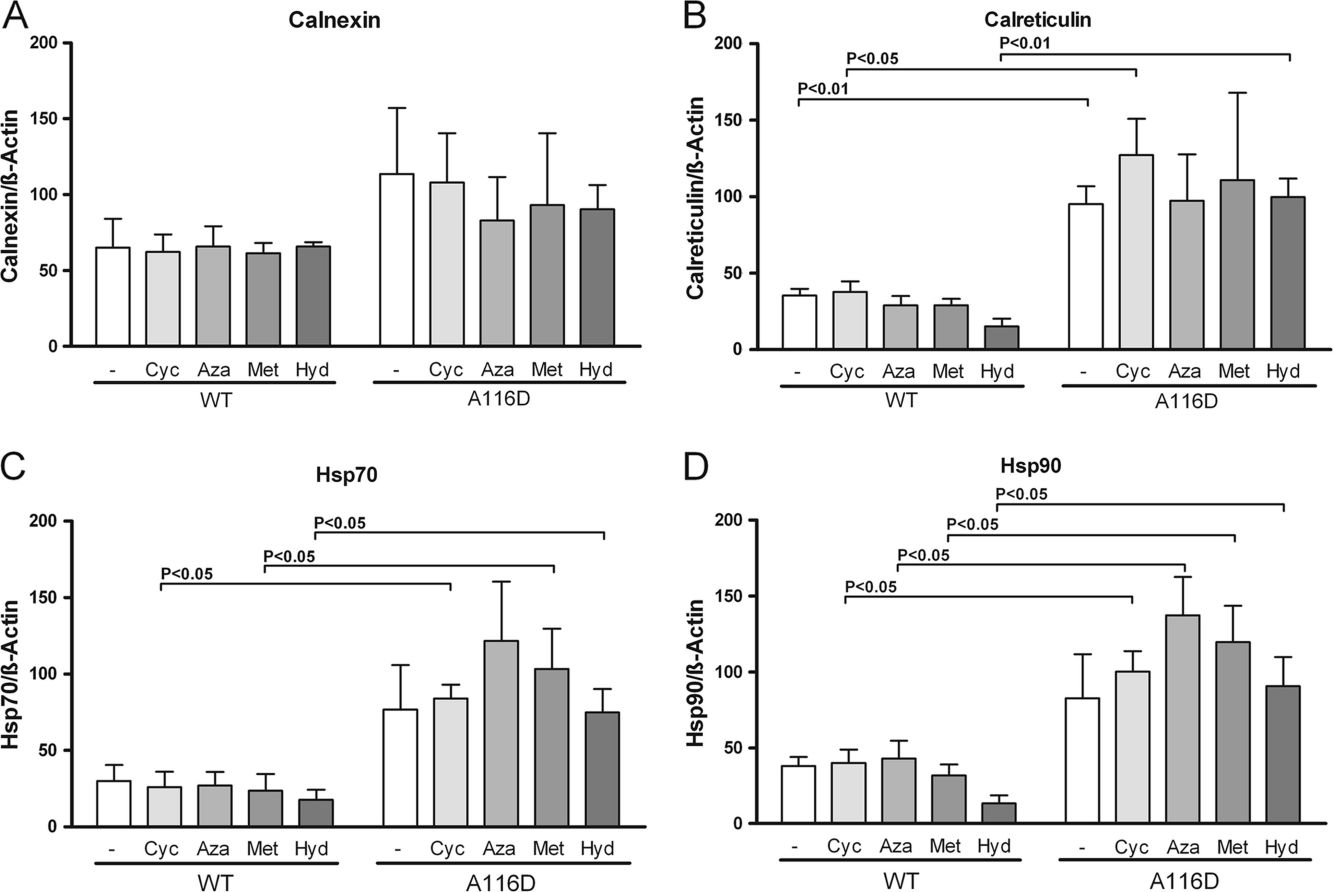
**Modulation of chaperone levels in the cells expressing SP-C^WT ^and SP-^A116D ^by pharmacologic substances**. Shown is the expression relative to β-actin of the chaperone proteins calnexin (A), calreticulin (B), Hsp70 (C) and Hsp90 (D) in MLE-12 cells expressing proSP-C^WT ^or proSP-C^A116D ^and either untreated (-) or exposed to cyclophosphamide (Cyc), azathioprine (Aza), methylprednisolone (Met), or hydroxychloroquine (Hyd). Only significant p-values are presented.

We determined the change in protein level of the two heat shock proteins Hsp90 and Hsp70, and the two ER-resident chaperones calreticulin and calnexin, in MLE-12 cells expressing SP-C^WT ^and SP-C^A116D^, after exposure to pharmacological substances used in ILD therapy: cyclophosphamide, azathioprine, methylprednisolone or hydroxychloroquine (Figure 4A-D). While in the case of calnexin no significant alterations were seen, cells expressing SP-C^A116D ^showed an increase in the expression of calreticulin, Hsp70 and Hsp90 at baseline and after treatment with pharmaceuticals. However, none of the applied drugs resulted in a significant change in expression level of any chaperone, neither in cells expressing SP-C^WT ^nor in cells expressing SP-C^A116D^. However, we noted a slight increase in the expression levels of Hsp70 and Hsp90 in SP-C^A116D ^cells treated with azathioprine (Figure 4C+D).

### Alterations in the intracellular lipid composition and composition of secreted lipids due to expression of SP-C^A116D ^and their response to pharmacological treatment

**Figure 5:**
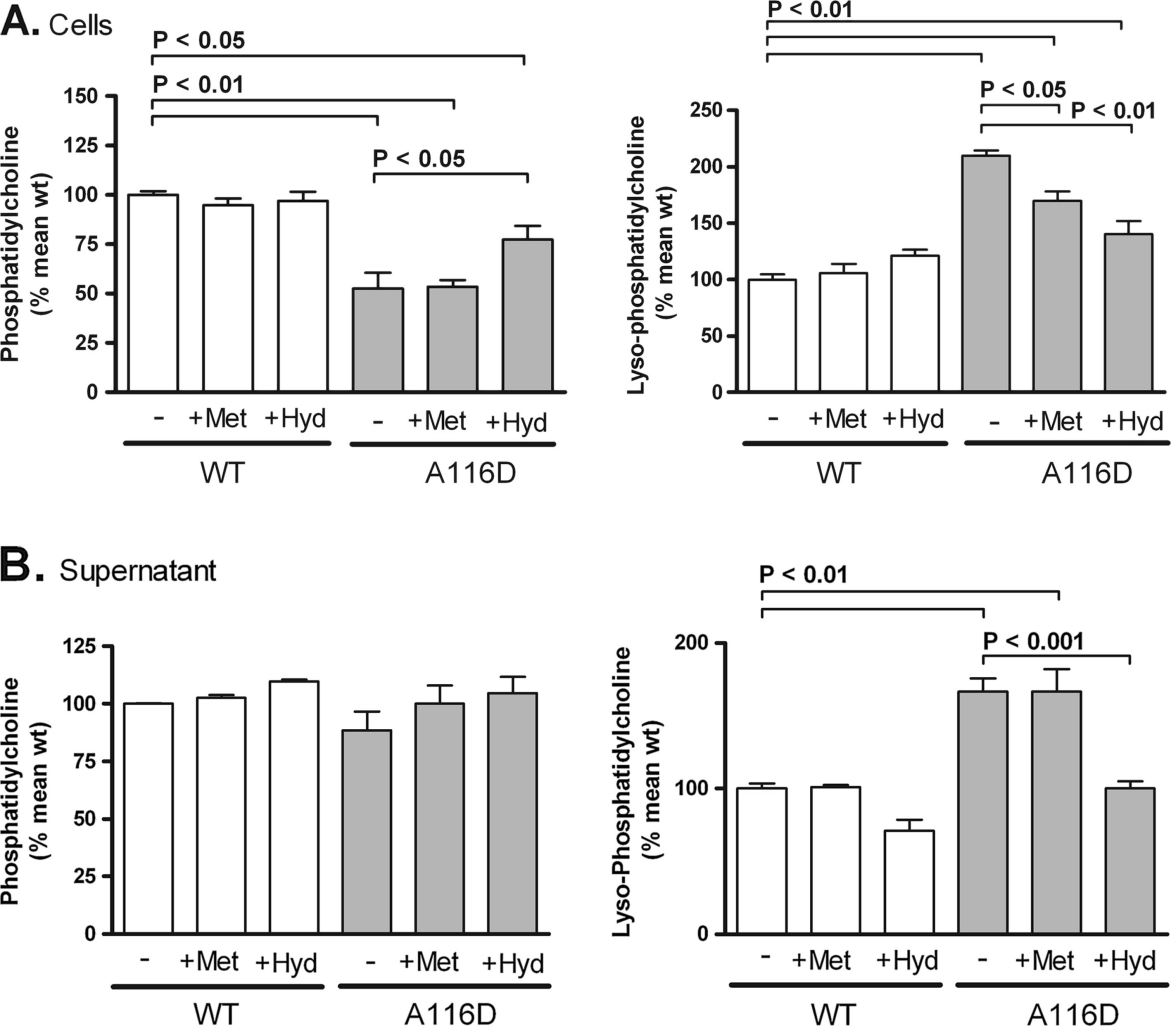
**Intracellular lipid content and lipid secretion of MLE-12 cells expressing SP-C^WT ^or SP-C^A116D^**. (A) Intracellular lipid content of cells stably expressing WT SP-C or A116D mutant were quantified by mass spectrometry. Untreated cells (-) or cells treated with 10 μM methylprednisolone (+Met) or hydroxychloroquine (+Hyd) for 24 hours prior to sample isolation. Values were calculated as % of the mean of the untreated WT values. (B) After removal of detached cells, the lipids in the cell supernatant were analyzed and presented as in (A). The graphs show relative amounts of phosphatidylcholine and lyso-phosphatidylcholine. Only significant p-values are depicted.

Mass spectrometric lipid analysis showed that total phospholipid amount was reduced in cell lysates from MLE-12 cells transfected with SP-C^A116D^, compared to cells transfected with SP-C^WT ^(Table 1). Moreover, the phospholipid composition was significantly altered: the percentage of phosphatidylcholine (PC) and ceramide (Cer) were significantly decreased while the percentages of lyso-phosphatidylcholine (LPC), phosphatidylserine (PS), phosphatidylethanolamine (PE) and sphingomyelin (SPM) were significantly increased in cells expressing SP-C^A116D^. Treatment with methylprednisolone or hydroxychloroquine did not correct the loss of PC in SP-C^A116D ^expressing cells, although a significant increase of PC was noted in the cells treated with hydroxychloroquine (Figure 5A, left chart). Treatment with methylprednisolone as well as with hydroxychloroquine led to a significant reduction of increased LPC levels in cells expressing SP-C^A116D ^(Figure 5A, right chart). The effect was more pronounced in the case of hydroxychloroquine.

**Table 1:** Phospholipid profile of transfected MLE-12 cells expressing mutant SP-C^A116D^

	Cell lysate			Supernatant		
	**WT**	**A116D**	**P**	**WT**	**A116D**	**P**

Total phospholipids (nmol/mg protein)	152.80 ± 9.6	126.50 ± 10.6	0.0334	20.57 ± 3.8	12.90 ± 6.2	0.0259

**Phospholipid classes (% of total PL)**						

Phosphatidylcholine	57.80 ± 1.0	52.80 ± 0.2	< 0.001	44.70 ± 0.1	38.73 ± 0.6	< 0.001

Lyso-Phosphatidylcholine	0.60 ± 0.1	0.90 ± 0.2	0.0171	5.90 ± 0.4	16.78 ± 1.3	0.001

Phosphatidylglycerol	0.30 ± 0.0	0.30 ± 0.0	ns	0.20 ± 0.1	0.10 ± 0.1	ns

Sphingomyelin	6.20 ± 0.3	6.90 ± 0.3	0.0061	8.03 ± 0.2	11.14 ± 0.2	< 0.0001

Ceramide	1.80 ± 0.1	1.60 ± 0.1	0.0133	3.30 ± 0.3	2.15 ± 0.2	0.0034

Glucosyl-Ceramide	0.10 ± 0.0	0.20 ± 0.0	ns	0.08 ± 0.0	0.09 ± 0.0	ns

Phosphatidylethanolamine	11.20 ± 0.5	14.50 ± 0.1	< 0.001	6.54 ± 0.2	6.53 ± 0.1	ns

Phosphatidylserine	6.50 ± 0.1	7.70 ± 0.3	< 0.001	11.71 ± 0.8	8.57 ± 0.1	0.0028

Data are means ± standard deviation from three independent experiments each performed in duplicate. ns = not significant

The phospholipid secretion by MLE-12 cells was assessed in the supernatant (Table 1). Similar to the intracellular lipid pattern, PC and Cer were significantly decreased in the supernatant of SP-C^A116D ^cells, while the percentages of LPC and SPM were increased. In contrast to the findings in cell lysate, PE was not altered in the supernatant of SP-C^A116D ^cells. Remarkably, PS was significantly decreased in the supernatant while being increased in cell lysate of SP-C^A116D ^cells. PC levels in the supernatant of cells treated with methylprednisolone were elevated compared to untreated cells by tendency, in WT as well as in A116D cells. A significant increase in LPC secretion was noted in cells expressing SP-C^A116D ^(Table 1; Figure 5B, right chart). Treatment with hydroxychloroquine, but not with methylprednisolone, restored LPC level in the supernatant of mutant SP-C expressing cells to the level observed in WT cells. Interestingly, hydroxychloroquine also reduced LPC in the supernatant of WT cells, albeit not significantly. Taken together, hydroxychloroquine treatment seems to be able to counteract the alterations of PC and LPC levels seen in cells expressing SP-C^A116D^.

### MLE-12 cells expressing SP-C^A116D ^secrete factors that stimulate surface expression of CCR-2 and CXCR-1 on CD4+ lymphocytes and of CXCR-1 on neutrophils

**Figure 6:**
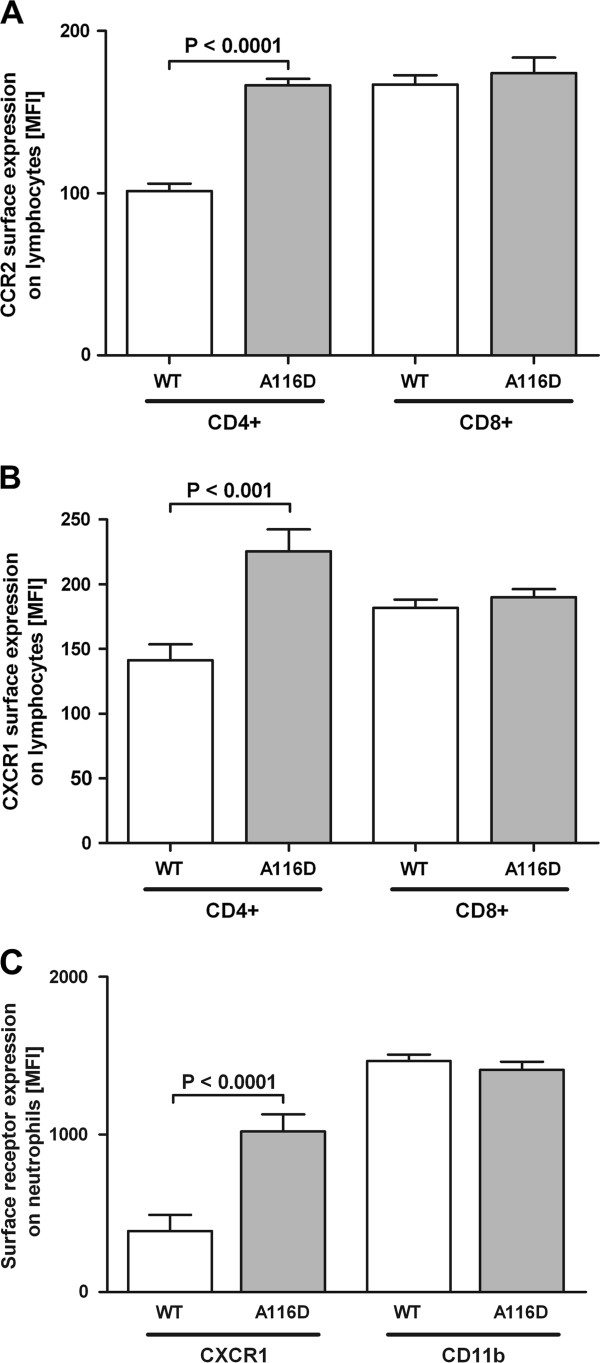
**Surface receptor expression on human lymphocytes and neutrophils**. Neutrophils and lymphocytes were isolated from the whole blood of different human donors and incubated with 7-fold concentrated supernatants obtained from MLE-12 cells expressing SP-C^WT ^or SP-C^A116D ^prior to flow cytometry analysis. Non-concentrated supernatants gave the same results, although less pronounced with a clear concentration dependency of the effects (data not shown). The receptor levels on the surface of lymphocytes after incubation with antibodies directed against (A) CCR2 or (B) CXCR1 are shown and expressed as mean fluorescence intensity (MFI). Another second marker-specific antibody was applied to distinguish between CD4+ and CD8+ lymphocytes. (C) The levels of CXCR1 and CD11b on isolated neutrophils. Significant changes are depicted with the corresponding p-values.

We hypothesized that accumulation of misfolded SP-C might somehow lead to an enhanced accumulation of leukocytes and thus boost the inflammatory reaction. To test this hypothesis, we examined whether cells expressing SP-C^A116D ^stimulated the expression of CCR2 on lymphocytes and CXCR1 on neutrophils by incubating isolated neutrophils or lymphocytes with 7-fold concentrated supernatants of MLE-12 cells expressing either WT or A116D SP-C. While no difference in surface receptor expression between WT and mutant was observed in CD8+ lymphocytes, CD4+ lymphocytes showed a highly significant increase in the level of surface receptor CCR2 expression in response to the supernatant of SP-C^A116D ^expressing cells (Figure 6A). The same was true in the case of CXCR1, which was increased on CD4+ lymphocytes after incubation with the mutant cell supernatant, but remained unaltered on CD8+ lymphocytes (Figure 6B). We further analyzed the surface receptor expression on neutrophils. The supernatant of cells expressing SP-C^A116D ^increased CXCR1 expression on neutrophils, but did not affect CD11b levels (Figure 6C). Non-concentrated supernatants gave the same results by tendency, although less pronounced. A clear concentration dependency of the effects was observed (data not shown). This suggests that SP-C^A116D^-expressing MLE-12 cells were able to modulate the surface receptor expression on the cells of immune system through the secretion of soluble factors into the medium.

## Discussion

Mutations in the *SFTPC *gene are a known cause of surfactant deficiency and very variable genetic ILD in children and adults. We investigated the consequences of the expression of SP-C with the mutation A116D for cell homeostasis and signaling in stably transfected MLE-12 cells. We further elucidated the ability of pharmaceutical drugs used in ILD therapy to modulate some of the cellular consequences of SP-C deficiency caused by the A116D mutation.

Stable transfection of MLE-12 cells with SP-C^A116D ^led to the intracellular accumulation of proSP-C^A116D ^processing intermediates which were not found in cells transfected with proSP-C^WT ^(Figure 1). However, the observed species resembled those seen in another SP-C mutation, I73T, *in vitro *as well as in BAL fluids from patients carrying this particular mutation [38]. The accumulation we observed in the case of A116D may thus reflect alterations in folding, trafficking and/or processing of proSP-C^A116D^. The first step in proSP-C processing is a cleavage at the C-terminal end [4]. Using an EGFP tag fused to the C-terminus of proSP-C showed no difference in processing intermediates of proSP-C^WT ^and proSP-C^A116D ^(Figure 1, right). This suggests that the mutation does not interfere with the first cleavage step that takes place at the C-terminus. Furthermore, this finding implies that the mutation does not abrogate the export from the ER and Golgi completely, because this cleavage occurs after trafficking through these compartments [4]. It is not known how the A116D mutation affects the folding of proSP-C, but subtle changes in conformation may be responsible for the appearance of a processing intermediate of approx. 17 kDa (Figure 1, band #3). A similar intermediate can be found in the BAL fluid of patients with the SP-C^I73T ^mutation, suggesting that this proSP-C form is being secreted from AT2 cells along with the mature SP-C that is produced by AT2 cells regardless of the presence of the I73T mutation [38].

Immunofluorescence assay of stably transfected MLE-12 showed that proSP-C^A116D ^often colocalized with EEA1 positive vesicles (Figure 2A). Early endosomes generally contain material that is taken up by endocytosis and is either recycled or routed for degradation [39]. Up to 80% of secreted lung surfactant is known to be reinternalized by AT2 cells from alveolar space [6]. Colocalization of proSP-C^A116D ^with EEA1 would thus imply that mutant protein is secreted together with surfactant and subsequently taken up again. On the other hand, while proSP-C^WT ^colocalized with syntaxin 2, proSP-C^A116D ^rarely did so. Syntaxin 2 is a SNARE protein involved in the secretion of lung surfactant, found in the plasma membrane and lamellar bodies of AT2 cells. Surfactant secretion is dependent on the fusion of lamellar bodies with the plasma membrane, which requires the activity of SNARE proteins. The lack of colocalization with Syntaxin 2 implies that mutant SP-C mostly does not reach lamellar bodies. Our results thus suggest that while physiological proSP-C forms are secreted via lamellar body fusion with the plasma membrane, proSP-C^A116D ^might take a different route and eventually gets lysosomally degraded.

The expression of mutated proteins frequently results in elevated cell stress. This has been shown for the SP-C BRICHOS domain mutations L188Q and Δexon4 [14,19]. We found that the constitutive expression of SP-C^A116D ^also significantly increased cell stress, compared to expression of SP-C^WT^. Likewise, the treatment with azathioprine resulted in an elevated stress level in cells expressing SP-C^WT ^as well as in cells expressing the mutant SP-C form where it further exacerbated cell stress due to the mutated SP-C. No significant alteration of cell stress was observed when cells were treated with methylprednisolone, hydroxychloroquine or cyclophosphamide. It can thus be concluded that, similar to other BRICHOS domain mutations, SP-C^A116D ^leads to elevated cell stress that cannot be corrected by the ILD drugs currently applied. Quite the contrary, our data suggest that some substances used in ILD therapy, especially azathioprine, pose potent stress factors *per se*.

After demonstrating that SP-C^A116D ^expression increases cell vulnerability to pharmacological stress stimuli, we further aimed to investigate the underlying intracellular mechanisms. Chaperone proteins assist in protein folding and are also involved in the folding of aberrantly processed proteins. Their synthesis is increased by cells as part of a cytoprotective mechanism to cope with increased intracellular stress and accumulation of misfolded proteins [27,40]. Still, without pharmacological boost, such cytoprotective mechanisms may not always be sufficient to normalize the cell function and maintain production of bioactive surfactant with a physiological lipid/protein composition. We investigated the influence of ILD drugs on the expression of four important chaperones in WT and A116D cells. The expression of Hsp70, Hsp90 and calreticulin was markedly increased in cells expressing SP-C^A116D ^(Figure 4A-D). Only in the case of calnexin no significant increase was observed (Figure 4A). No drug did affect the expression of any chaperone in a significant fashion in WT cells. Furthermore, no drug was able to further increase the elevated chaperone levels seen in cells expressing A116D. However, azathioprine and methylprednisolone led to a slight, albeit not significant, increase in the expression of Hsp70 and Hsp90 by A116D cells. These findings imply that the drugs we tested did not possess the ability to significantly increase chaperone levels. It is thus questionable whether they exert their beneficial effects in ILD treatment by enhancing ER folding capacity.

The packaging and secretion of lung surfactant lipids is very closely linked to the expression of the hydrophobic surfactant proteins in AT2 cells [6]. The lipid composition of stably transfected MLE-12 cells was similar to that previously described for human fetal AT2 cells, especially with regard to PC composition [41]. In the cells expressing SP-C^A116D ^we found a pronounced drop of total cellular PC, while LPC was increased (Figure 5A, Table 1). It is known that PC is degraded to LPC by an intrinsic phospholipase A2-like activity, and that LPC is toxic to various cells [42]. LPC is a known inhibitor of the lung surfactant activity and has the ability to penetrate directly into interfacial films to impair lowering of the alveolar surface tension during dynamic compression [43,44]. Elevated LPC levels in the SP-C^A116D^-expressing cells could also explain the heightened sensitivity towards exogenous stress described above. Generation of LPC cannot account for the decrease of PC mass in SP-C^A116D ^expressing cells, but additional factors, which directly interfere with the synthesis and packaging of PC, must also be responsible.

Among phospholipids secreted by cells expressing the A116D mutation into culture supernatant, PC was again decreased by 14% and LPC was increased by 184%, in accordance with a reduced surfactant function [41,43]. Treatment with hydroxychloroquine ameliorated the increase in intracellular and secreted LPC and decrease in secreted PC, but did not completely correct it (Figure 5). While methylprednisolone treatment resulted in a reduction of intracellular LPC, it did not have an effect on PC levels. The capacity of the treatment with methylprednisolone and hydroxychloroquine to correct the lipid disturbances caused by the A116D mutation likely represents one of the mechanisms by which these treatments are empirically helpful in some patients with A116D mutations [28] (own unpublished results). However, their mode of action still remains a conundrum and needs to be investigated further.

Injury of lung epithelial cells resulting from endogenous or exogenous stress leads to accumulation of pulmonary leukocytes and finally to inflammation and tissue remodeling. Leukocytes are attracted to sites of inflammation along a chemokine gradient. This chemotaxis is mediated via chemokine receptors on the surface of leukocytes [45]. In previous studies we demonstrated that, among a plethora of chemokine receptors involved in this network, specifically CCR2 on lymphocytes and CXCR1 on neutrophils modulate pulmonary immunity in human inflammatory lung diseases [37,46]. We found that cells expressing SP-C^A116D ^released soluble factors into the medium that increase surface expression of CCR2 and CXCR1 on CD4+ lymphocytes and CXCR1 on neutrophils (Figure 6A-C). When activated, the high affinity IL-8 receptor CXCR1 mediates antibacterial killing capacity [46,47]. Increases in surface expression levels of CCR2 and CXCR1, respectively, might have the potential to modulate the pulmonary immune response with regard to antibacterial (CXCR1) and profibrotic (CCR2) responses [46,48]. However, the soluble factors involved in the induction of chemokine receptor expression as well as the functional consequences of this phenomenon remain to be addressed in future studies.

There are some limitations to our study. A potential limitation is that our system represents a homozygous mutation rather than a heterozygous *SFTPC *mutation where one functional allele is still present. However, endogenous SP-C is expressed in the MLE-12 cells [29]. Although expression of exogenous SP-C from the CMV promoter present on the plasmid vector is likely higher, both forms are present. Given that all known patients with SP-C mutations are heterozygous carriers, expressing one copy of the wild type gene, the experimental model closely reflects the *in vivo *condition. A second important limitation to consider is that one has to be careful when extrapolating findings in MLE-12 cells in culture to the actual alveolar environment. In particular the compositional profile of lipids found in cell culture supernatant is not necessarily representative for surfactant lipids secreted by AT2 cells in vivo as cells in culture may secrete lipids by mechanisms other than via lamellar bodies. Finally, the A116D mutation seems to be rare and some of its features may be specific and not generalizable.

## Conclusion

We showed impaired proSP-C processing, elevated cellular stress and unfavorable changes of the surfactant lipid composition in a murine alveolar epithelial cell line expressing mutant SP-C with the BRICHOS domain mutation A116D. Some of the cellular aspects of disease we demonstrated could be modulated with drugs used in the therapy of ILD patients, thus providing insight into their potential therapeutic mode of action. We also showed that MLE-12 cells expressing SP-C^A116D ^secrete certain soluble factors that likely promote chemotaxis of immune cells and may thereby exaggerate inflammatory and fibrotic processes. Therefore, our study adds to understanding of the effects that *SFTPC *mutations impose on alveolar epithelial cell biology and pave the way for a more precise pharmacological targeting in patients with SP-C deficiency.

## Abbreviations

SP-C: surfactant protein C; SFTPC: surfactant protein C gen; SP-B: surfactant protein B; ILD: interstitial lung disease; BAL: bronchial lavage; AT2: alveolar type 2; SNARE: SNAP receptors; SNAP-23: soluble NSF attachment protein 23; EGFP: enhanced green fluorescent protein; HA: hemagglutinin; EEA1: early endosomal antigen 1; HRP: horseradish peroxidase; LDH: lactate dehydrogenase; ER: endoplasmic reticulum; HSP70/90: heath shock protein 70/90; PC: phosphatidylcholine; LPC: lysophosphatidylcholine; PE: phosphatidylethanolamine; PS: phosphatidylserine; SPM: sphingomyelin; PG: phosphatidylglycerol; CXCR1: CXC chemokine receptor; CCR2: chemokine C-C motif receptor 2; FITC: fluorescein isothiocyanate; PE2: phycoerythrin; MFI: mean fluorescence intensity; Cyc: cyclophosphamide; Aza: azathioprine; Met: methylprednisolone; Hyd: hydroxychloroquine.

## Competing interests

The authors declare that they have no competing interests.

## Authors' contributions

RZ, MW, MG and SK conceived and designed the study as well as analyzed and interpreted the data. CS, MW, SK, TT and EK performed the experiments with exception of MS lipid analyses, performed by GL and GS, and flow cytometry analyses, performed by AH and DH. RZ and MG wrote the paper. All authors read and approved the final manuscript.

## Authors' information

This paper contains parts of the doctoral thesis of Christiane Sparr. Until 2009 Sunčana Kern published as Sunčana Moslavac.

## Pre-publication history

The pre-publication history for this paper can be accessed here:

http://www.biomedcentral.com/1471-2466/12/15/prepub
